# IGF-1 drives chromogranin A secretion *via* activation of Arf1 in human neuroendocrine tumour cells

**DOI:** 10.1111/jcmm.12473

**Published:** 2015-03-08

**Authors:** Christin Münzberg, Katharina Höhn, Denis Krndija, Ulrike Maaß, Detlef K Bartsch, Emily P Slater, Franz Oswald, Paul Walther, Thomas Seufferlein, Götz von Wichert

**Affiliations:** a Department of Internal Medicine I, University of UlmUlm, Germany; b Central Facility for Electron Microscopy, University of UlmUlm, Germany; c Department of Visceral, Thoracic and Vascular Surgery, Philipps University MarburgMarburg, Germany

**Keywords:** ADP-ribosylation factor, neuroendocrine secretion, insulin-like growth factor 1, carcinoid syndrome, Golgi apparatus

## Abstract

Hypersecretion is the major symptom of functional neuroendocrine tumours. The mechanisms that contribute to this excessive secretion of hormones are still elusive. A key event in secretion is the exit of secretory products from the Golgi apparatus. ADP-ribosylation factor (Arf) GTPases are known to control vesicle budding and trafficking, and have a leading function in the regulation of formation of secretory granula at the Golgi. Here, we show that Arf1 is the predominant Arf protein family member expressed in the neuroendocrine pancreatic tumour cell lines BON and QGP-1. In BON cells Arf1 colocalizes with Golgi markers as well as chromogranin A, and shows significant basal activity. The inhibition of Arf1 activity or expression significantly impaired secretion of chromogranin A. Furthermore, we show that the insulin-like growth factor 1 (IGF-1), a major regulator of growth and secretion in BON cells, induces Arf1 activity. We found that activation of Arf1 upon IGF-1 receptor stimulation is mediated by MEK/ERK signalling pathway in BON and QGP-1 cells. Moreover, the activity of Arf1 in BON cells is mediated by autocrinely secreted IGF-1, and concomitantly, autocrine IGF1 secretion is maintained by Arf1 activity. In summary, our data indicate an important regulatory role for Arf1 at the Golgi in hypersecretion in neuroendocrine cancer cells.

## Introduction

Carcinoid tumours of the gastrointestinal tract are rare neuroendocrine tumours (NETs) that are increasing in incidence. These tumours are derived from neuroectodermal cells and are predominantly found in the gastrointestinal tract. When these tumours secrete a variety of hormones, they are classified as functional NETs. This condition is associated with the clinical symptoms of flush, diarrhoea and hypertension and constitutes the so-called carcinoid syndrome that substantially affects the quality of life of patients suffering from this disease. BON cells have been established from a human pancreatic carcinoid tumour and are the best characterized model so far to study the biology of human NETs *in vitro* 1. We have demonstrated previously that growth of BON cells is largely regulated by autocrinely secreted insulin-like growth factor-1 (IGF-1), which is present in many NETs 2,3.

Various stimuli have been identified that induce neuroendocrine secretion in these cells, including members of the PKC 4 and PKD family 5. It becomes more and more evident that a key event in hormone secretion is the exit of secretory products from the Golgi apparatus. However, the precise intracellular mechanisms that are responsible for the excessive exit of secretory granules from the *trans*-Golgi network (TGN) are still elusive. Small GTPases of the Rho and Arf families have been characterized as key players regulating this process 6. Based on their sequence homology, human Arf proteins are classified into three classes: class I (Arf1 and Arf3), class II (Arf4 and Arf5) and class III (Arf6) 7. Arf-GTPases cycle between an inactive GDP-bound form and an active GTP-bound form through the action of guanine nucleotide exchange factors (GEFs) and GTPase-activating proteins (GAPs). The large number of these regulatory proteins compared with the relatively small number of Arfs suggests that activation of these GTPases is under extensive regulatory control under normal circumstances. Arf1, as a class I Arf, is classically associated with the Golgi apparatus to regulate vesicle trafficking where it interacts with a number of proteins including Arf GEFs and GAPs, certain enzymes involved in lipid metabolism and protein adaptors that aid in the recruitment of specific cargos 6.

A role for Arf-GTPases in cancer has only recently been suggested 8. Arf GTPases have been shown to be active regulators of proliferative and/or invasive properties of cancer cells 9,10 and their function in invasion may stem from their role at the crossroad between membrane trafficking, recycling and Rho- mediated actin remodelling 11,12. Inhibition of Arf activity by Brefeldin A (BFA) causes a rapid but reversible disruption of the Golgi apparatus leading to the vesiculation of the *cis*-Golgi and *trans*-Golgi network (TGN) 13. Interestingly, BFA treatment resulted in tumour growth inhibition *in vitro* and *in vivo* 14. Surprisingly, it is still not clear whether the disruption of the TGN is required for the anti-proliferative effect 15,16. However, one could speculate that the loss of autocrinely secreted growth factors significantly contributes to the impaired growth. Given the fact that carcinoid tumours are typically associated with hypersecretion and that autocrine stimuli contribute to the hypersecretion phenotype, it is reasonable to assume that Arf-GTPases could play an important role in NETs biology.

Here we show that class I Arfs are the predominant subfamily expressed in neuroendocrine BON cells, where Arf1 colocalizes with chromogranin A in a perinuclear region. Interestingly, inhibition of Arf1 activity or expression significantly impaired neuroendocrine secretion. In addition, we show for the first time that growth factors such as IGF-1 can induce constitutive activity of Arf1 *via* an IGF-1 receptor (IGFR)/MEK-dependent signal transduction pathway. Moreover, constitutive activity of Arf1 is facilitated by autocrinely secreted IGF-1, which in turn is maintained by constitutive activity of Arf1 indicating a positive feedback loop.

## Materials and methods

### Cell culture and transfection

Human BON carcinoid tumour cells were authenticated in February 2014, QGP-1 in December 2012 by Leibniz-Institut DSMZ GmbH (Braunschweig). BON cells were maintained in DMEM, QGP-1 cells in RPMI 1640 (Invitrogen, Karlsruhe, Germany) supplemented with 10% (v/v) foetal bovine serum (Biochrom AG, Berlin, Germany) and 1% (v/v) Penicillin-Streptomycin (Invitrogen) in a humidified atmosphere of 5% CO_2_: 95% air at 37°C and passaged every 4 days. Nanofectin Transfection Kit (PAA, Toronto, ON, Canada) was used for transfection of BON cells.

### DNA constructs

Arf1mRuby was generated by PCR, using a GST-Arf1 construct (described previously 17) as a template with a forward primer (5′-GCGGTACCATGGGGAACATCTTCGCC-3′) and a reverse primer (5′-GCCTCGAGCTTCTGGTTCCGGAGCTG-3′). The PCR fragment was inserted into pcDNA3-mRuby. Arf1(T31N)mRuby was created using the above set of primers and pXS-Arf1(T31N)-HA (a kind gift from Julie Donaldson, NHLBI, USA) as a PCR template. All DNA constructs were verified by DNA sequencing.

### Antibodies and reagents

Monoclonal anti-Arf1 (clone ARFS 1A9/5) were purchased from Santa Cruz Biotechnology Inc. (Santa Cruz, CA, USA), anti-ARF4 (11673-1-AP) from Proteintech (Manchester, UK), anti-ARF5 (clone 1B4) from Abnova (Jhongli, Taiwan), anti-Arf3 (clone 41), anti-GS28 (611184) from BD Biosciences (Franklin Lakes, NJ, USA), anti-Human chromogranin A (A0430) from DakoCytomation (Glostrup, Denmark), anti-ARF6 (ab77581), anti-IGF-1 (ab9572) and anti-beta COP (ab2899) from Abcam (Cambridge, UK), anti-Golgin-97 (CDF4), Alexa-Fluor-488/568/647 labelled anti-mouse or anti-rabbit IgG were purchased from Invitrogen. Anti-β-Actin (clone AC-15) were purchased from Sigma-Aldrich, Steinheim, Germany, anti-Phospho-IGF-IR (Tyr1161), anti-IGF-IRβ (c-20), anti-ERK2 (C-14) from Santa Cruz Biotechnology Inc., anti-Phospho-Akt (Ser473) (D9E), anti-Akt (pan) (C67E7), anti-Phopho-p44/42 MAPK (Thr202/Tyr 204) (197G2), anti-Phospho-p70 S6 Kinase (Ser371), anti-p70 S6 Kinase (49D7) from Cell Signaling Technology (Millipore, Billerica, MA, USA). Enhanced chemiluminescence (ECL) detection reagents were purchased from GE Healthcare (Buckingamshire, UK). Brefeldin A (5 μg/ml), LY-294,002 (20 μM), PD98059 (20 μM) and DMSO were purchased from Sigma-Aldrich, BMS-536924 (10 μM), MK-2206 (5 μM), Rapamycin (20 ng/ml) from Selleck Chemicals (Houston, TX, USA) and IGF-1 (50 ng/ml) from Invitrogen; final concentrations in brackets. All other reagents were at the highest grade available.

### RNA interference

siRNA targeting Arf1 (5′-CACCATAGGCTTCAACGTGGA-3′), Arf3 (5′-CTCCTTGTCTTTGCAAACAAA-3′) and a negative control siRNA were purchased from Qiagen (Hamburg, Germany) and used in a final concentration of 30 nM. siRNA transfections were performed with the HiPerfect Transfection Reagent (Qiagen), according to the manufacturer's instructions. Briefly, BON cells were plated, the medium was changed after 24 hrs medium and the transfection mix was added. The medium was changed again the next day and cells were transfected once more. The efficiency of Arf1/Arf3 knockdown was validated by western blotting and qRT-PCR.

### Quantitative real-time PCR

RNAs were extracted from cells using QiaZol (Qiagen), treated with DNase I and purified with RNeasy kit (Qiagen), all according to the manufacturer's instructions. cDNAs were prepared from total RNAs using Superscript reverse transcriptase (Invitrogen). The quantitative RT-PCR (qRT-PCR) for detection of Arf1 to Arf6 was performed with a QuantiTect Primer Assay—Hs_ARF1_1_SG, Hs_ARF3_1_SG, Hs_ARF4_1_SG, Hs_ARF5_1_SG and Hs_ARF6_2_SG, respectively (Qiagen), according to the manufacturer's protocol. The house keeper actin was detected by the forward primer (5′-GACGTGGCAGAGAAGTACCTG-3′) and the reverse primer (5′-GGGCAGTTCCAACGATGT-3′).

### Western blotting

BON cells transfected with negative, Arf1 or Arf3 siRNA were scraped and lysed on ice. Lysis buffer contained 150 mM NaCl, 50 mM Tris-Cl pH 7.5 and 1% (v/v) IGEPAL CA-630 (Sigma-Aldrich), supplemented with a protease and posphatase inhibitor cocktail (Roche, Mannheim, Germany). Whole cell lysates were denaturated in 6× reducing SDS-Sample Buffer (Boston BioProducts, Ashland, MA, USA), resolved by SDS-PAGE and further analysed using standard methods.

### Growth curve

BON cells were seeded in 12-well plates (Greiner Bio-one, Frickenhausen, Germany) and cultured under normal conditions. After 3 days medium was changed and serum free medium, supplemented with inhibitors, was added. Every second day medium supplemented with inhibitors was changed again. Cells were counted after 96 hrs and cell number was calculated from triplets.

### Active-Arf pull-down assays

A GST-fused GGA1-GAT construct 18 was used to retrieve the active Arf from cell lysates. GST fusion proteins were purified from transformed BL21 cells induced with 1 mM IPTG for 2 hrs at 37°C. Bacteria were lysed with bacterial lysis buffer [20 mM Tris, 250 mM NaCl, 2.5 mM MgCl_2_, lysozyme (Sigma-Aldrich) 1 mg/ml] and centrifuged, supernatants were incubated with glutathione sepharose beads (GE Healthcare) overnight at 4°C. The binding of the GST-GGA1 to the beads was confirmed in Coomassie staining (Fig. S1). BON cells were serum starved for 12 hrs, subsequently treated with different inhibitors, IGF-1 or DMSO for 4 hrs and afterwards lysed with RIPA buffer [0.5% (v/v) IGEPAL CA-630, 0.5% (w/v) Sodium deoxycholate, 0.1% (w/v) SDS, 150 mM NaCl, 50 mM HEPES (Invitrogen), 2.5 mM MgCl_2_, 10% (v/v) Glycerol (Sigma-Aldrich)]. The extracts were incubated with equal amounts of the GST fusion proteins immobilized on sepharose beads for 2 hrs at 4°C. After extensive washing, bound proteins were eluted with SDS-sample buffer, resolved by SDS-PAGE and analysed by western blotting. The intensity of detected bands was quantified *via* Image J.

### Chromogranin A ELISA

BON cells were seeded in 6-well-plates and pre-treated with inhibitors in 1% FCS/DMEM for 12 hrs, except BFA and BMS that were pre-treated for 4 hrs. After washing with DPBS cells were incubated with inhibitors in serum-free DMEM for 2 hrs again. QGP-1 cells were pre-treated with inhibitors for 12 hrs in 10% FCS/RPMI and after washing with DPBS incubated with inhibitors in serum-free RPMI for 6 hrs. Supernatants were collected and coated in 96-well-Maxisorp plates (Nunc, Wiesbaden, Germany) over night at 4°C. Chromogranin A was detected by the antibody from DAKO. The signal from horseradish peroxidase-conjugated secondary antibody was detected using O-Phenyldiamine dihydrochloride substrate (Sigma-Aldrich). The reaction was stopped with 2 M H_2_SO_4_ and optical density was measured at 490 nm.

### IGF-1 ELISA

BON cells were pre-treated with inhibitors in 1% FCS/DMEM for 6 hrs. After washing with DPBS cells were incubated with inhibitors in 1% FCS/DMEM for 4 hrs again. The IGF-1 levels from supernatant were measured using the Quantikine Human IGF-I Immunoassay from R&D Systems (Minneapolis, MN, USA). IGF-1 levels from the culture medium were subtracted from all samples after measurement of the optical density.

### Immunocytochemistry

BON cells were seeded on Poly-l-Lysine-coated coverglasses. After fixation and permeabilization, the coverslips were incubated with primary- and secondary antibodies for 1 hr at RT. Samples were mounted in ProLong Gold antifade reagent with or without containing the counterstain DAPI (Invitrogen). Images were acquired using an inverted microscope (Olympus, IX71, 100×; Olympus, Hamburg, Germany) connected to a CCD camera (Orca-HR, Hamamatsu, Japan) or by confocal microscopy on a LSM 710 from Zeiss (Jena, Germany). Overlay images were obtained using ImageJ.

### Tissue sections and immunohistochemistry

Tissue sections from formalin fixed and paraffin embedded archived tumour samples of six insulinomas and three gastrinomas were obtained from the tissue bank of the Department of Pathology, Philipps University of Marburg. All investigations and all patient material in this study were assessed under a research protocol approved by the Philipps University of Marburg Ethics Committee (No. 104/99). Patients gave their informed consent in written form. The slices with a thickness of 4 μm were deparaffinized with xylene and rehydrated through a graded alcohol series. Demasking of antigens was performed in citrate buffer (pH 6.0) in microwave after the blockade of endogenous peroxydases by 3% H_2_O_2_. Unspecific binding sites were blocked by 2% BSA and primary antibody was incubated over night at 4°C. After washing secondary antibody was incubated for 30 min. at RT. After removal of unbound antibody ABC complex (Vectastain Elite ABC Kit; Vector Laboratories, Burlingame, CA, USA) was added and incubated for 30 min. After washing slices were incubated with Nova Red (Vector Laboratories) where after counterstaining with Harris hematoxylin (Sigma-Aldrich) was performed. Embedded sections were imaged at Keyence Biorevo BZ-9000 (Keyence, Neu-Isenburg, Germany).

### Electron microscopy

BON cells were seeded on carbon-coated, glow-discharged sapphire discs (50 or 160 μm in thickness, diameter 3.05 mm, Engineering Office M. Wohlwend GmbH, Sennwald, Switzerland). siRNA transfection was carried out as described above. The cells grown on sapphire discs were high pressure frozen according to two different protocols as described in Höhn *et al*. 2011. For both protocols freeze substitution and Epon embedding was performed as described in 19 with a substitution medium consisting of acetone with 0.2% osmium tetroxide, 0.1% uranyl acetate and 5% of water for good contrast of the membranes. For TEM data thin sections with a thickness of 70 nm were cut with a Reichert Ultracut microtome and imaged with a JEOL 1400 TEM (JEOL Ltd., Tokyo, Japan).

### Statistics

Graphs and determination of statistical significance of results were prepared by the use of GraphPad Prism 5 (GraphPad software Inc., La Jolla, CA, USA). Significance was tested by one-way anova with Dunnett′s Multiple Comparison post-test.

## Results

### Arf1 is the predominant Arf isoform in NET cells and partially colocalizes with chromogranin A positive vesicles

**Figure 1:**
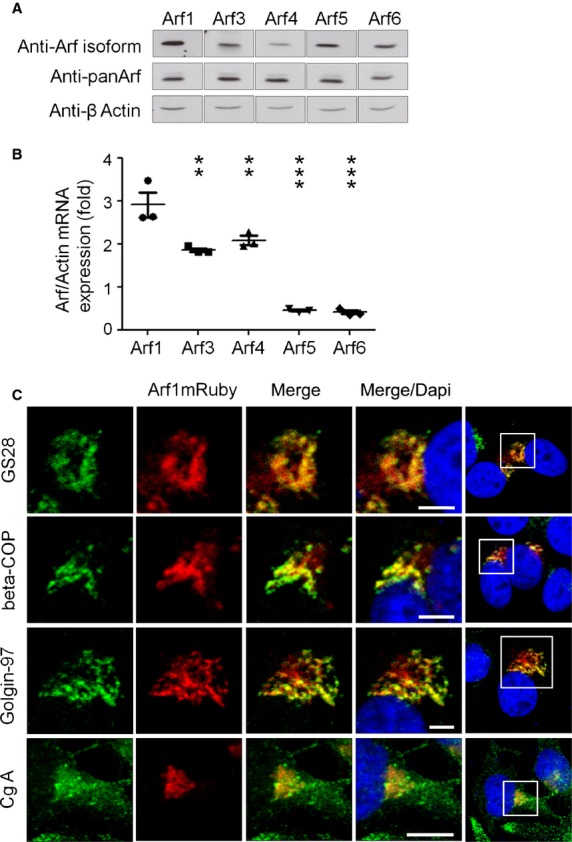
Arf1 is the predominat Arf isoform in BON cells and partially colocalizes with chromogranin A positive vesicles. (A) Expression of the five human Arf proteins in BON cell lysates detected by isoform-specific antibodies in Western Blot analysis. The total Arf protein was detected by anti-panArf antibody, actin levels by anti-β actin antibody. (B) Expression levels of Arf mRNAs in untreated BON cells were measured by quantitative RT-PCR analysis (±SEM, *n* = 3; ***P < 0.001; **P < 0.01). (C) Arf1 localizes to *cis*- and *trans*-Golgi compartments and partially colocalizes with chromogranin A-postive neuroendocrine vesicles in BON cells. BON cells were transfected with mRuby tagged Arf1 wild-type. Fixed cells were stained for specified markers (GS28, beta COP, Golgin-97, chromogranin A) as well as DAPI, and images were acquired using confocal microscope; scale bar: 5 μm.

First we wanted to analyse the expression and subcellular distribution of Arfs in human neuroendocrine cells. Therefore, we separated BON cell lysate and detected the different Arf proteins by isoform-specific antibodies in Western Blots. We found all five human Arf proteins to be expressed in BON cells (Fig.1A). To quantitatively analyse the expression of different Arf isoforms, we performed quantitative real-time PCR. After extraction of total mRNA, the expression levels of different Arf isoforms were calculated according to the expression of actin in these cells. Interestingly, Arf1 was the most abundantly expressed Arf isoform (Fig.1B). Results were confirmed in a second NET cell line (QGP-1 cells; Fig. S5A and B). Since Arf1 has been shown to be an important regulator of the recruitment of coat proteins to Golgi membranes, we analysed the subcellular distribution of Arf1 in these cells. Due to the lack of suitable Arf1-antibodies for immunostaining we transiently transfected BON cells with an mRuby-tagged wild type Arf1 (Arf1-mRuby) and analysed it's distribution by confocal microscopy (Fig.1C). As expected, Arf1-mRuby colocalized with the cis-Golgi markers GS28 and beta-COP as well as with Golgin-97, which is localized at the trans-site of the Golgi network. To address a possible association with the compartment that contains neuroendocrine secretion products, cells were again transfected with Arf1-mRuby and stained for chromogranin A (CgA), an established marker for neuroendocrine vesicles. Indeed, Arf1mRuby partially colocalized with neuroendocrine vesicles in BON cells. Taken together these data suggest that Arf1 is the predominant Arf-isoform in neuroendocrine BON cells and that its association with neuroendocrine vesicles may suggest a function for Arf1 in deregulated secretion of NETs (expression of Arf1 in samples of pancreatic human NET samples was substantiated by immunohistochemistry for Arf1 expression in samples of pancreatic insulinomas and gastrinomas; Fig. S6).

### Inhibition of Arf1 activity leads to loss of neuroendocrine vesicles

**Figure 2:**
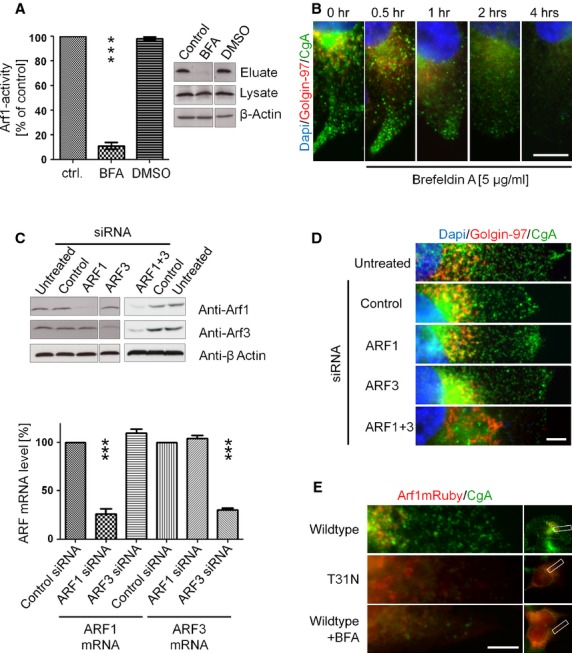
Arf1 is constitutively active and inhibition of Arf1 leads to loss of neuroendocrine vesicles. (A) Brefeldin A, DMSO or untreated control cells were lysed and lysates incubated with GST-GGA1-GAT beads. The retained Arf1-GTP, Arf1 input and actin levels were assessed by western blotting (±SEM, *n* = 4–5; ***P < 0.001). (B) Brefeldin A treatment caused the loss of neuroendocrine granula. BFA treated and control cells were fixed and stained for chromogranin A/Alexa 488, Golgin-97/Alexa 568 and DAPI. Pictures show sections regions from BON cells; scale bar: 5 μm. (C) siRNA mediated knock-down of Arf1 and Arf3 was quantified after 48 hrs in qRT-PCR (±SEM, *n* = 3; ***P < 0.001) and also confirmed in western blot by the use of subtype-specific antibodies. (D) Double knock-down of Arf1 and Arf3 by RNA inference caused a loss of neuroendocrine granula, observed after immunostaining of chromogranin A/Alexa 488, Golgin-97/Alexa568 and DAPI by fluorescence microscopy. Pictures show sections regions from BON cells; scale bar: 5 μm. (E) Transfection of dominant negative Arf1(T31N)mRuby revealed a loss of chromogranin A containing vesicles comparable to BFA treated and Arf1mRuby wildtype transfected BON cells. Fluorescence microscopy shows sections regions of chromogranin A/Alexa 488 stained BON cells; scale bar: 5 μm.

Neuroendocrine tumour cells are constantly secreting chromogranin A into the supernatant. We therefore asked the question whether there is a contribution of Arf1 activity to deregulated secretion of chromogranin A in BON cells. However, until now a role of the activation of Arf1 in neuroendocrine cells has not been addressed. The GAT domains of the clathrin adaptors GGA1, GGA2 and GGA3 were shown to interact with GTP-bound Arf 20. Using GGA1-GAT as bait, we were able to determine the level of activated, GTP-bound Arf1 in neuroendocrine BON cells. Indeed, GST-GGA1-GAT bound significant amounts of active Arf1 from untreated cell lysates (Fig.2A). Arf1 activation can be blocked by the fungal metabolite Brefeldin A (BFA), which targets a subset of Arf guanine nucleotide exchange factors (Arf GEFs), preventing the exchange of GDP to GTP. As expected, after 4 hrs of incubation with BFA, the cells lost more than 90% of active Arf1 (Fig.2A). In good agreement with the proposed function of Arf1, we observed the loss of neuroendocrine granula in BON cells upon treatment with BFA (Fig.2B). To further examine whether these effects are due to the loss of function of Arf1, and to address possible off target effects of BFA, we performed RNA interference experiments. The efficiency of siRNA-mediated knock-down of Arf1 and Arf3 was confirmed in western blot analysis and also by quantitative real-time analysis (Fig.2C). As expected by previous reports, due to the high degree of homology and functional redundancy among class I Arfs 21, the single isoform-selective (Arf1 or Arf3) knockdown did not mimic the effect of BFA treatment. As anticipated, the double-knockdown of Arf1 and Arf3 completely depleted neuroendocrine vesicles from the cytoplasm (Fig.2D). To further address the importance of Arf1 activity, we transfected the cells with a dominant negative mutant of Arf1, Arf1-T31N (Fig.2E). Indeed, dominant inhibition of Arf1 activity imitated the phenotype of BFA treatment. Due to redundant functions of Arf1 and Arf3 in the control of neuroendocrine secretion our data identifies these class I Arfs as key regulators in the maintenance of neuroendocrine hypersecretion.

### Arf1 activity is required to maintain Golgi structure in neuroendocrine BON cells

**Figure 3:**
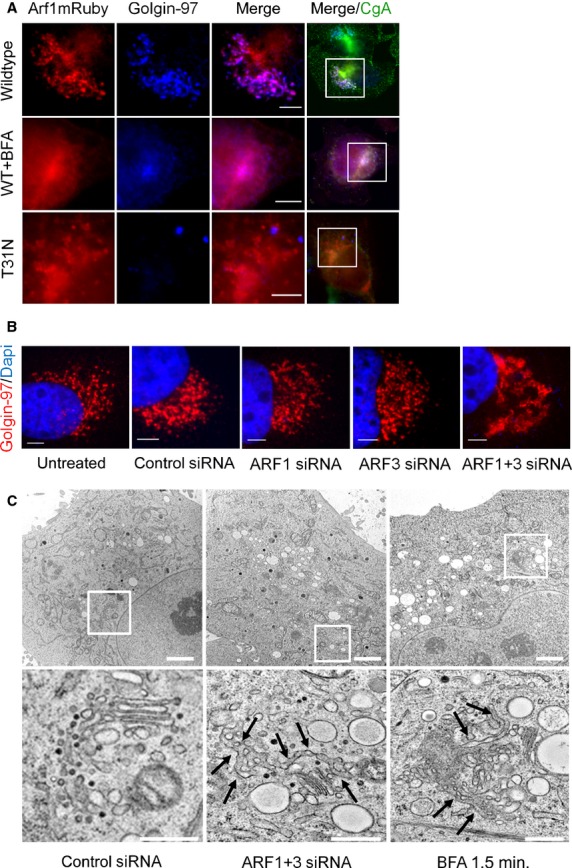
Constitutive activity of Arf1 is required to maintain Golgi structure in neuroendocrine BON cells. (A) BON cells transfected with wildtype or dominant negative mutant (T31N) of Arf1mRuby were fixed and stained for Golgin-97/Alexa647 and chromogranin A/Alexa488. Additionally, wildtype transfected cells were treated with BFA for 4 hrs. Fluorescence microscopy shows enlarged sections of the Golgi region of BON cells; scale bar: 5 μm. (B) SiRNA transfected BON cells were stained for Golgin-97/Alexa568 and DAPI after fixation. Fluorescence microscopy shows enlarged sections of the Golgi region; scale bar: 1 μm. (C) Electron microscopy of siRNA transfected or BFA treated BON cells. Golgi regions were enlarged; scale bar: 2 μm, enlarged regions 500 nm.

To further characterize the function of Arf1 in neuroendocrine secretion, we analysed the structure of the Golgi apparatus in BON cells transfected with wild type mRuby-tagged Arf1. Immunofluorescence microscopy revealed finely structured and stacked Golgi morphology, and Arf1 colocalized well with Golgin-97 (Fig.3A, upper panel). However, after incubation of cells with BFA, Arf1 lost its distinct localization and appeared diffuse in the cytoplasm of the cells. In good agreement, Golgin-97 also appeared diffuse after BFA treatment indicating the disruption of the Golgi apparatus (middle panel). The expression of the dominant negative mutant of Arf1 caused an effect similar to the BFA treatment. In fact, Arf1(T31N)-mRuby-expressing cells showed a diffuse localization of Arf1 in the cytoplasm as well as the loss of a structured Golgi morphology (bottom panel). Interestingly, BON cells transfected with either Arf1 or Arf3 siRNA had normal Golgi morphology, similar to untransfected or control siRNA transfected cells. However, in accordance with our previous results, inhibition of the expression of both class I Arfs by transfection with specific siRNAs led to a phenotype similar to the treatment with BFA (Fig.3B). In the double knockdown cells the Golgi appeared swollen and displayed large tubes. To analyse this in greater detail, we employed electron microscopy (Fig.3C). In sharp contrast with the finely structured Golgi apparatus of control cells, after siRNA-mediated knockdown of Arf1 and Arf3, we observed large tubes, emanating from the remaining Golgi stacks and spreading throughout the cytoplasm. In addition, a similar effect was obtained in BFA-treated cells (Fig.3C). Taken together, these results strongly suggest that expression and constitutive activity of class I Arfs is required to maintain Golgi structure in these tumour cells.

### Activity of Arf1 is regulated by activation of the IGF-1 receptor and following MEK/ERK intracellular signalling

**Figure 4:**
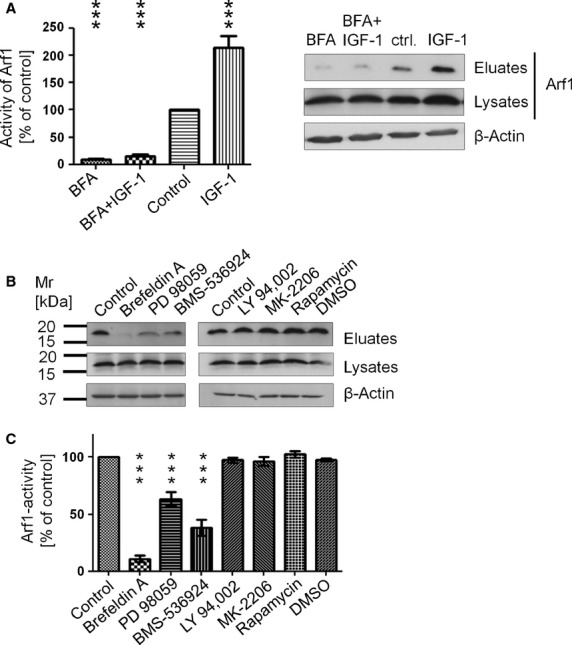
Activity of Arf1 is regulated by activation of the IGF-1 receptor and following MEK/ERK intracellular signalling. (A) Activity of Arf1 after 4 hrs of IGF-1 stimulation in BON cells was assessed in pull-down assays, followed by western blot analysis of the eluates (±SEM, *n* = 4; ***P < 0.001). (B) BON cells treated with different inhibitors of the IGF-1 signalling pathway were lysed and the activity of Arf1 was measured in a pull-down assays. Therefore, cell lysates were incubated with GST-GGA1-GAT beads and retained Arf1-GTP and Arf1 input levels were afterwards assessed by western blotting (±SEM, *n* = 4; ***P < 0.001).

**Figure 5:**
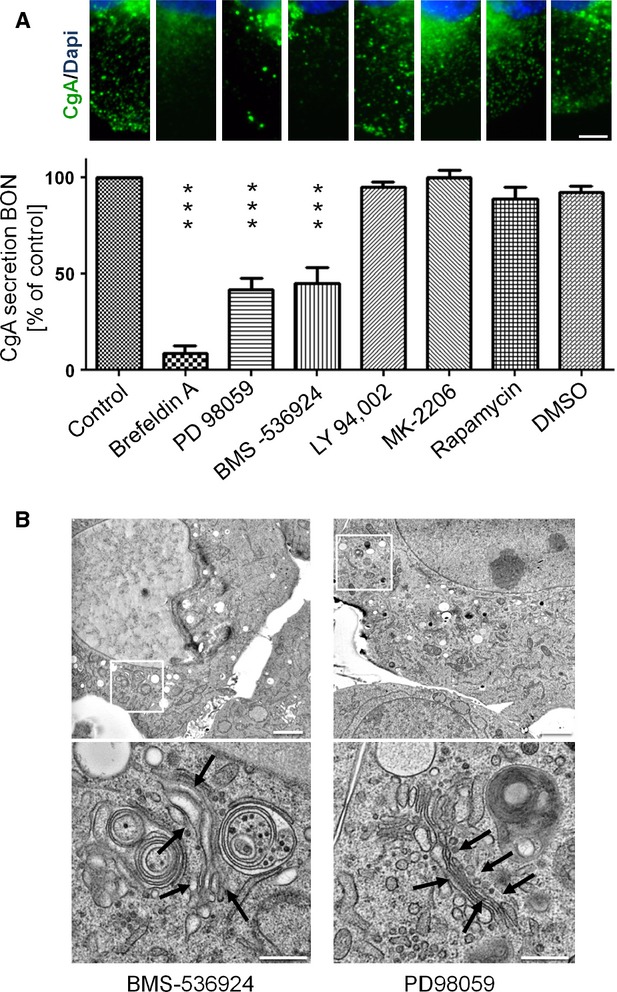
An IGF-1/MEK/ARF pathway controls neuroendocrine secretion and Golgi morphology in BON cells. (A) Neuroendocrine secretion of untreated control cells or inhibitor treated BON cells was analysed in fluorescence microscopy after fixation and immunostaining of chromogranin A/Alexa488 and DAPI. Pictures show sections regions from BON cells; scale bar: 5 μm. Secretion of chromogranin A was furthermore quantified by ELISA, where supernatants of inhibitor treated BON cells or untreated control cells were measured (±SEM, *n* = 4; ***P < 0.001). (B) Electron microscopy of BMS-536924 or PD98059 treated BON cells showed loss of neurendocrine granula. Golgi regions were enlarged; scale bar: 2 μm, enlarged regions 500 nm.

Since para- and autocrine factors are known to induce constitutive activation of signal transduction pathways in cancer cells, it was attractive to speculate that growth factor-dependent signals could mediate constitutive activity of Arf1. We and others 2,3,22 have previously shown that the IGF-1 is a potent autocrine stimulus in neuroendocrine cancer cells and that neuroendocrine secretion at least in part depends on IGF-1. Therefore, we wanted to address whether IGF-1 dependent signalling led to the high constitutive activity of Arf1 in BON cells. Indeed, treatment of BON cells with IGF-1 induced pronounced activation of Arf1. In sharp contrast, treatment with BFA inhibited basal Arf1 activity and prevented the IGF-1 induced stimulation of Arf1 in these cells (Fig.4A). To further analyse the IGF-1-dependent effects, we treated BON cells with specific inhibitors of either the IGFR or known downstream targets of IGF-1 signalling (Fig.4B). The cells were treated with the IGF-1 receptor inhibitor BMS-536924, the PI3K inhibitor LY249,002, the AKT inhibitor MK-2206, the mTOR inhibitor Rapamycin or the ERK inhibitor PD98059. The functionality of the inhibitors was confirmed in western blot analysis (Fig. S2). Cell lysates of pre-treated cells were used to perform GGA1-GAT pull-down assays and the amount of active Arf1 was subsequently determined in western blot analysis. Inhibition of IGF1R and ERK kinase activity led to a significant decrease in Arf1 activation (Fig.4B). In contrast, inhibition of the PI3K/AKT/mTOR signalling pathway did not show any reduction in the activity of Arf1 (Fig.4B). These data suggest that IGF-1-induced intracellular signal transduction *via* the MEK/ERK cascade is required to maintain activity of Arf1. In good agreement, cells treated with BMS-536924 or PD89059 showed a significantly lower content of neuroendocrine vesicles as observed by immunostaining for CgA (Fig.5A top). The IGFR/MEK pathway as well as activity of Arf1 were required to maintain neuroendocrine secretion as determined by the secretion of CgA into the supernatant (Fig.5A bottom). The important results for the inhibition of neuroendocrine secretion by inhibition of Arf1 *via* a IGFR/MEK pathway were confirmed in the QGP-1 cell line (Fig. S5C). In good agreement with our previous results, analysis of the Golgi morphology by electron microscopy of cells treated with IGF1R and ERK inhibitors partially recapitulates aspects of the BFA inhibition and Arf1 and 3 mediated siRNA knockdown. Arrows indicated morphological changes in Golgi stacks *e.g*. in tube size and morphology in BMS-536924 treated cells, which show similarities in morphology to the BFA-treatment and Arf1 and 3 siRNA double knockdown samples (Fig.5B). Taken together these data imply that autocrine and/or paracrine IGF-1 is the major upstream initiator of constitutive Arf1 activity *via* the MEK/ERK pathway.

### Arf1 regulates secretion of IGF-1 and is required for anchorage-dependent growth

**Figure 6:**
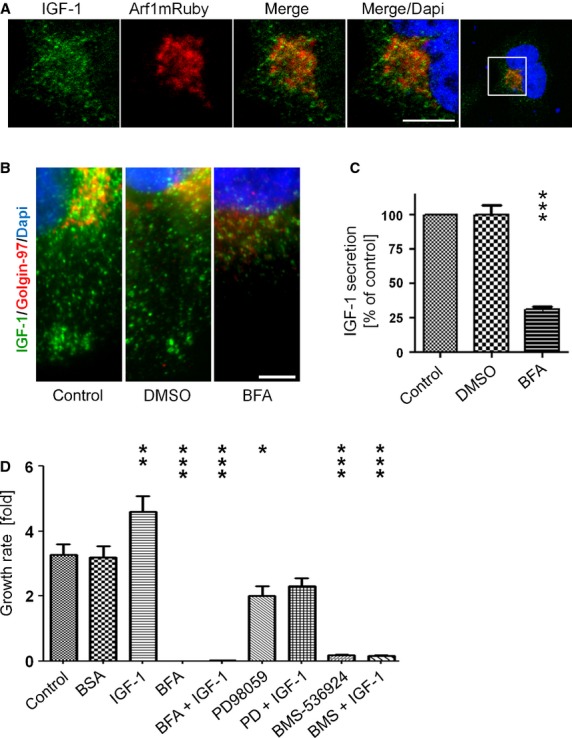
Arf1 regulates secretion of IGF-1 and is required for anchorage dependent growth. (A) Arf1mRuby transfected BON cells were fixed, stained for IGF-1/Alexa488 and DAPI and analysed in confocal microscopy; scale bar: 5 μm. (B) BFA treated BON cells and untreated control cells were fixed and IGF1/Alexa488, Golgin-97/Alexa568 and DAPI were detected after immunostaining by fluorescence microscopy. Pictures show sections regions from BON cells; scale bar: 5 μm. (C) IGF-1 secretion from supernatants of untreated control, Brefeldin A or DMSO treated BON cells were quantified in ELISA (±SEM, *n* = 4; ***P < 0.001). (D) Growth curve analysis of untreated, BSA, IGF-1 or inhibitor treated BON cells. Cell numbers were counted after 4 days (±SEM; *n* = 3 from triplicates).

Insulin-like growth factor-1 is a known secretion product of BON cells and has been shown to be a potent stimulator of growth and secretion in these cells. Since IGF-1 drives neuroendocrine secretion, it was attractive to speculate that IGF-1-dependent activation of Arf1 contributes in turn to autocrine secretion of IGF-1 as well. In good agreement with the data from the chromogranin A staining, IGF-1 partially colocalized with Arf1mRuby in a perinuclear compartment (Fig.6A). Treatment of cells with BFA induced a pronounced depletion of IGF-1-positive vesicles from the cytoplasm of these cells. Some residual IGF-1 was retained in the perinuclear compartment of the cells (Fig.6B). In accordance, quantification of the IGF-1 concentration in the supernatant showed a marked reduction of about 75% of IGF-1 secretion compared to control cells when measured in ELISAs (Fig.6C). Since IGF-1 has been shown to be a major mediator of anchorage-dependent growth, we were curious whether treatment with the established inhibitors would alter anchorage dependent growth in these cells. Indeed both, the IGF1R inhibitor BMS-536924 and BFA significantly inhibited the growth of BON cells under basal conditions and upon IGF-1 stimulation (Fig.6D, control of stimulation by IGF-1 see Fig. S3). In summary, these data imply that IGF-1 maintains its autocrine secretion by activation of Arf1. Furthermore, IGF-1 and Arf1 are strong regulators of anchorage dependent growth in BON cells.

## Discussion

Here, we showed that Arf1 is the predominant member of the subfamily of class I Arfs expressed in the NET cell lines BON and QGP-1. Inhibition of Arf1 activity or -expression significantly impaired neuroendocrine secretion. In addition, we showed for the first time that autocrinely secreted growth factors such as IGF-I can induce constitutive activity of Arf1 *via* an IGF1R/MEK dependent signal transduction pathway. It has been suggested that small GTPases such as Arf1 can contribute to the development and maintenance of a malignant phenotype 23. Arf1 is known to be involved in several important cellular processes, including membrane trafficking and activation of phospholipase D 24. Recently Arf1 has been implicated in the control of cell proliferation, due to its ability to regulate pRB/E2F1 activity and gene expression enhancing proliferation and progression of breast cancer 25. Inhibition of endogenous Arf1 expression resulted in the suppression of breast cancer cell migration and proliferation through activation of the phosphatidylinositol 3-kinase pathway 9. However, there is still an open debate whether the pro-malignant functions of Arf1 are exclusively mediated by Arf1-dependent control of the Golgi structure/function 15,16. Our data clearly suggest that the class I Arf-mediated control of the Golgi exit and subsequent supply of secretion products to the plasma membrane is a key event in the maintenance of hypersecretion in neuroendocrine cancer cells. These data are in good agreement with reports showing Arf1-dependent cellular exit of soluble proteins such as sVEGFR 26 or MMP-8 27 or supply of transmembrane proteins such as integrins 11 or growth factor receptors 28,29. At present we do not know whether Arf1 also regulates the availability of growth factor receptors on the cell surface in BON cells and further studies are required to address this question. Nonetheless, our data reveal that Arf1 is a potent regulator of neuroendocrine secretion and release of growth factors such as IGF-1. We have shown previously that IGF-1 is regulating growth, survival and secretion in BON cells in an autocrine fashion 2,3 and it has been suggested that growth factors such as the epidermal growth factor (EGF) induce activation of Arf1. Besides EGF, also VEGF/VEGFR2 signalling was shown to promote Arf1 activity 30. Our data identify IGF-1 for the first time as the main mediator of constitutive Arf1 activity in this NET model. Similarly, inhibition of IGF1 receptor signalling led to a reduction in Arf1 activation, which in turn reduced the secretion of chromogranin A. Although constitutive activity of Arf1 has been implicated by various studies, conclusive models for the maintenance of basal/constitutive Arf1 activity have been lacking to date. Our data convincingly show that IGF-1-dependent signal transduction is required for the Arf1-dependent release of chromogranin A as well as of IGF-1, which in turn stimulates the activation of Arf1. IGF-1R activation leads to activation of numerous signalling cascades. The main pathways activated through signal transduction of the IGF system are Ras/Raf/MEK/ERK and the PI3K/AKT signalling pathway 31–33. IGF-1-mediated activation of Arf1 required activation of MEK-1 but not PI3K/AKT in BON cells. These data are in good agreement with a previous study that identified MEK-1 activity as a prerequisite for the secretion of neurotensin from BON cells 34. Interestingly, recent studies have shown that MAPK signalling, namely the activation of ERK2, is instrumental for the early secretory pathway as shown for the secretion of transferrin and alpha1-antitrypsin 35,36. More specifically, the MAPK protein ERK2 was found to directly phosphorylate Sec16 on threonine residue 415, and this phosphorylation event controlled the number of ER exit sites (ERES) as well as ER-to-Golgi transport. The functional relevance of this regulation might lie in the fact that signalling by growth factors, such as IGF-1, induces protein synthesis and thereby increases the protein load exiting the ER. Phosphorylation of Sec16 by ERK2 leads to an increase of ERESs, thus enabling cells to cope with conditions of higher cargo flux 35. Moreover, a tight functional interaction between Arf1 and MAPK signalling has recently been suggested by a study showing that depletion of Arf1 attenuated adrenergic receptor-mediated activation of ERK1/2 without altering intracellular receptor trafficking, whereas expression of the constitutively active mutant Arf1Q71L and ARNO, a GDP-GTP exchange factor of Arf1, markedly enhanced the activation of Raf1, MEK1, and ERK1/2 37. Interestingly, previous studies have shown that the activity of BFA-sensitive Arf GEFs such as BIG1 and BIG2 can be controlled by phosphorylation. BIG1 and BIG2 have both been characterized as protein kinase A-anchoring proteins and the phosphorylation status regulated not only their GEF activity but also controlled their translocation to membranes, suggesting a possible role of BIGs in a crosstalk between Arf and PKA pathways 38. However, the precise role for MAPK-dependent signalling in the activation of Arf1 requires further analysis.

Taken together our data show that Arf1 has an important role in regulating Golgi structure and secretion in a model of neuroendocrine cancer.

## Supporting Information

**Figure S1** Expression and purity of GST-GGA1-GAT. **Figure S2** Validation of the used inhibitors. **Figure S3** Activation of IGF-1R by recombinant human IGF-1 in BON cells. **Figure S4** Inhibition of BON cell growth by different inhibitors. **Figure S5** Arf1 is the predominant Arf isoform in QGP-1 cells where Arf activity as well as IGF1R/ERK signalling regulate neuroendocrine secretion. **Figure S6** Expression of Arf1 in human neuroendocrine tumours of the pancreas.
