# Supplementary material for: IGF-1 drives chromogranin A secretion via activation of Arf1 in human neuroendocrine tumour cells
Source: J Cell Mol Med. 2015 Mar 8;19(5):948–59. doi: 10.1111/jcmm.12473 (PMC4420598; doi:10.1111/jcmm.12473)
Supplement: Supplementary file 1 [file jcmm0019-0948-sd1.docx]

***Supporting Information***


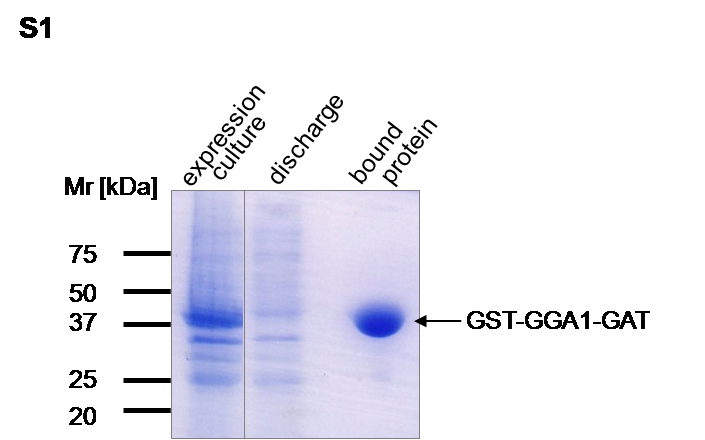


Figure S1 **Expression and purity of GST-GGA1-GAT.**

GST-GGA1-GAT was recombinantly expressed in *E. coli* and bacterial lysates were incubated with GSH-beads. The bound fusion protein was eluated from the beads and the purity was analyzed by Coomassie staining.


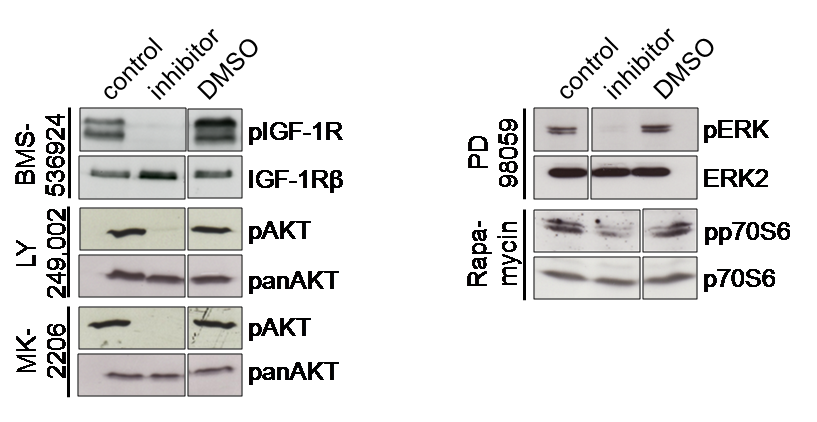


Figure S2 **Validation of the used inhibitors.**

The specificity of the varios inhibitors that were used in the assays was confirmed in western blot analysis by the use of phospho-specific antibodies.


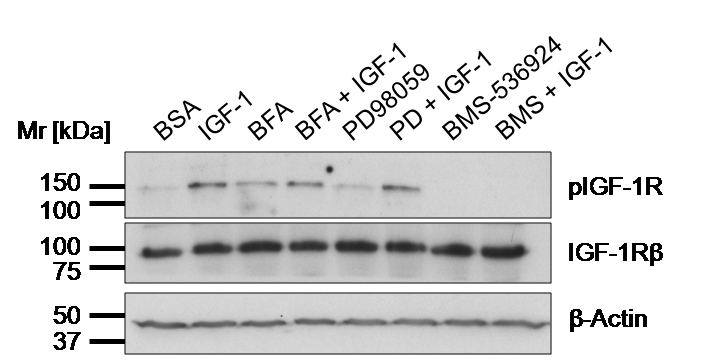


Figure S3 **Activation of IGF-1R by recombinant human IGF-1 in BON cells.**

The IGF-1R of serum-starved and inhibitor treated BON cells was activated by adding 50 ng/ml IGF-1 to the medium. IGF-1 was dissolved in 0.1 % BSA/ DPBS and the solvent was added as control. After 4 h cells were lysed and activation of the receptor was confirmed by western blotting using a phospho-specific IGF-1R antibody.


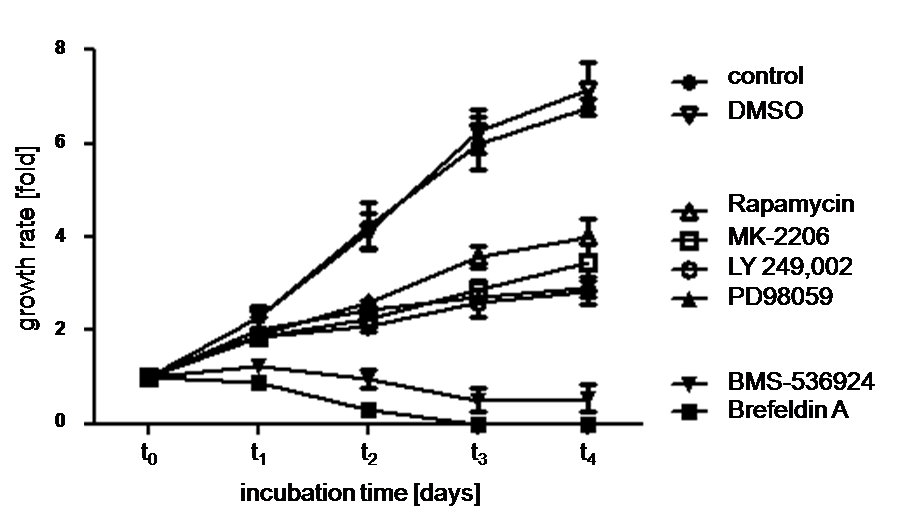


Figure S4 **Inhibition of** **BON cell growth by different inhibitors.**

Growth curve analysis of untreated (control), DMSO or inhibitor treated BON cells in the presence of 10% FCS in the culture medium. Cell number were counted every 24 h (± SEM; n=3 from triplicates). BMS-536924 was used in a concentration of 10 µM, Brefeldin A 5 µg/ml, LY-294,002 20 µM, PD98059 20 µM, MK-2206 5 µM and Rapamycin 20 ng/ml.


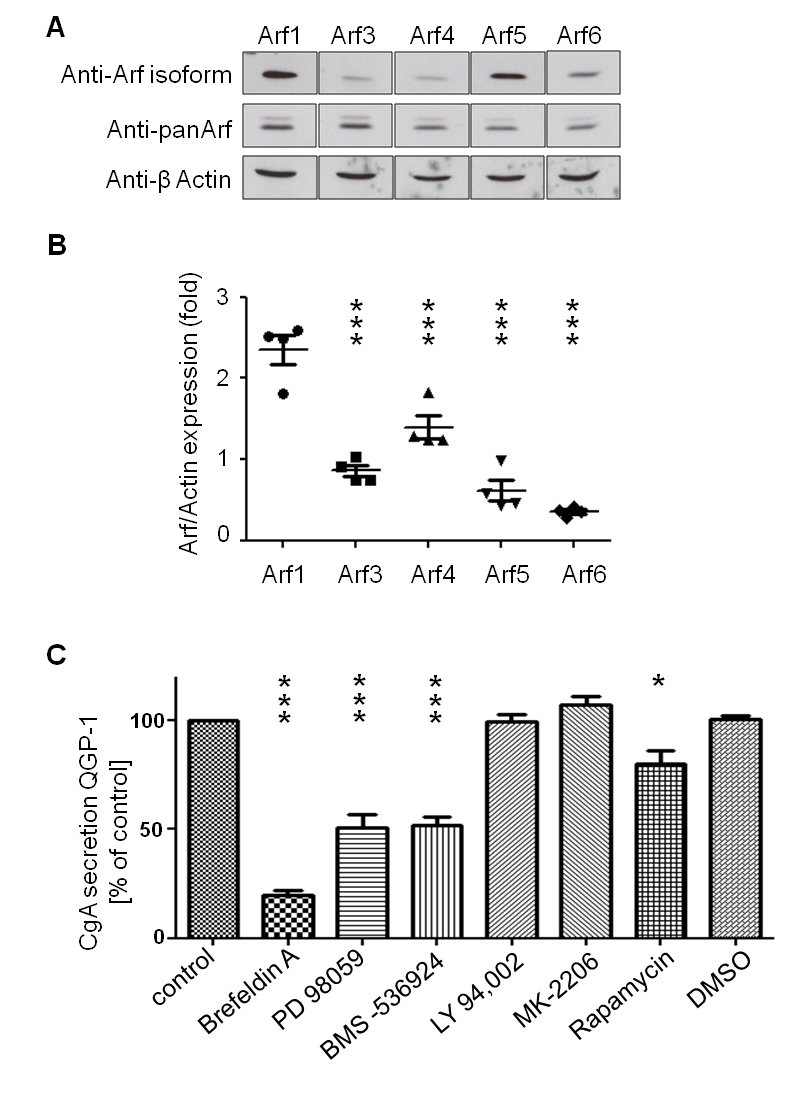


Figure S5 **Arf1 is the predominant Arf isoform in QGP-1 cells where Arf activity as well as IGF1R/ERK signaling regulate neuroendocrine secretion.**

(A) Expression of the single Arf proteins in QGP-1 cell lysate detected by isoform-specific antibodies in Western Blot analysis. The total Arf protein was detected by anti-panArf antibody, actin levels by anti-β actin antibody. (B) Expression levels of Arf mRNAs in untreated QGP-1 cells were measured by quantitative RT-PCR analysis. (± SEM, n=4; *** *P*<0.001). (C) Signaling pathways that control neuroendocrine secretion in QGP-1 cells where analyzed by measurement of secreted Chromogranin A. Therefore supernatants of inhibitor treated QGP-1 cells or untreated control cells were quantified in ELISA (± SEM, n=3; *** *P*<0.001, * *P*<0.1).


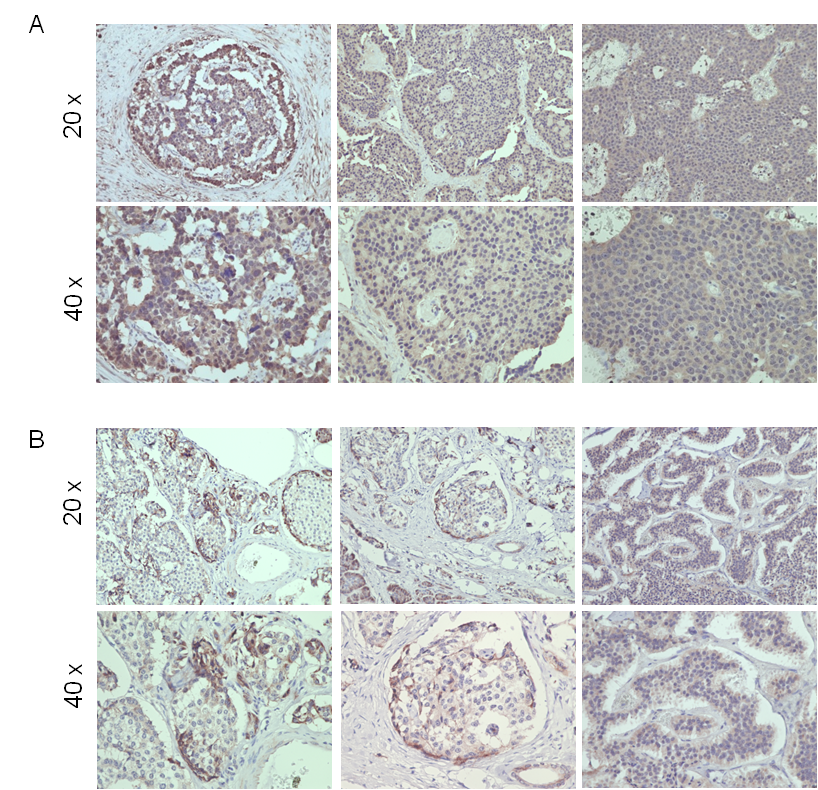


Figure S6 **Expression of** **Arf1 in human neuroendocrine tumors of the pancreas.**

Arf1 was detected in tissue sections of human neurendocrine tumors in immunohisto-chemistry by the use of the Arf1-specific antibody. Shown are representative sections from different Insulinoma (A) as well as Gastrinoma (B). Positive staining of Arf1 is indicated by the brown color. Arf1 was observed mainly in the neureondocrine tumor cells and barly in stromal cells.
